# Regionally specific picture naming benefits of focal tDCS are dependent on baseline performance in older adults

**DOI:** 10.1007/s11357-025-01674-x

**Published:** 2025-05-10

**Authors:** A. Yucel, F. Niemann, M. Meinzer, A. K. Martin

**Affiliations:** 1 School of Psychology, University of Kent, Canterbury, UK; 2 Department of Neurology, University Medicine Greifswald, Greifswald, Germany; 3 Kent Medway Medical School, Canterbury, UK

**Keywords:** Word retrieval, Non-invasive brain stimulation, Ageing, Baseline cognition, Picture naming, Left-lateralised language network

## Abstract

Word-finding difficulty is a common challenge in older age and is linked to various neuropathological conditions associated with ageing. Transcranial direct current stimulation (tDCS) has shown promise as a cognitive enhancement tool for both healthy aging and age- related cognitive disorders. However, its effectiveness in enhancing word-finding ability remains inconsistent, especially among healthy adults. Variability across studies is likely due to factors such as task selection, stimulation parameters, and small and variable sample sizes. Additionally, many studies have overlooked within-study variability of potentially relevant participant characteristics, including the role of baseline performance in evaluating tDCS efficacy. In this preregistered study, we examined 72 older and 72 younger adults using a double-blind, sham-controlled design, delivering anodal focal tDCS to either the left inferior frontal gyrus or the left temporoparietal junction. Baseline naming performance and fluid intelligence were measured before stimulation. Anodal stimulation of the left inferior frontal gyrus significantly increased response speed for object and action naming in older adults, but crucially only in older adults who performed poorly during the baseline naming session, *p* = .02, η^2^ₚ = 0.17. Findings demonstrate regionally specific effects of focal tDCS in healthy older individuals in greater need for naming facilitation. Notably, performance on a broad measure of fluid intelligence was unrelated to stimulation response, suggesting task specificity of this effect.

**Supplementary Information:**

The online version contains supplementary material available at 10.1007/s11357-025-01674-x.

## Introduction

Difficulty with word retrieval is a common complaint in older adults [17, 42] often exacerbated in pathological aging conditions [30]. This decline can significantly impact communication, quality of life, and functional independence [6]. As the populations in Western countries continue to age, there is an urgent need for effective interventions to mitigate cognitive decline and preserve language abilities in older adults [38]. One promising intervention is transcranial direct current stimulation (tDCS), a non-invasive technique that modulates neural excitability by delivering a weak electrical current to targeted brain regions [39]. TDCS has gained traction due to its portability, cost-effectiveness, and ease of use, making it a potential tool for cognitive enhancement and rehabilitation [4]. However, variability in baseline cognitive functioning among older adults introduces significant challenges in assessing the effects of tDCS. Individuals with higher baseline cognitive functioning often show less benefit from tDCS across various cognitive domains [20]. These baseline differences contribute to within-study variability. Moreover, between-study differences are often identified and can be attributed to factors such as small sample sizes, non-focal stimulation paradigms, and inconsistencies in stimulation parameters.

Despite its potential, existing literature on tDCS for word retrieval in older adults remains inconsistent. Some studies report improvements in word retrieval following stimulation of the left inferior frontal gyrus (IFG; [15, 23]) or left dorsolateral prefrontal cortex [8], but these studies often suffer from small sample sizes and a focus on single brain regions. Moreover, the impact of baseline cognitive performance, either in terms of word retrieval or general fluid intelligence, on tDCS responsiveness has not been systematically investigated. Evidence from other domains (e.g., word generation, visual detection) suggests that individuals with lower baseline performance may benefit most from stimulation [22, 31], although this remains unknown in relation to word retrieval. It is also important to ascertain which baseline measure is optimal to predict tDCS responsiveness. To the authors’ knowledge, this is also the first study to directly compare tDCS responsiveness in relation to baseline functioning in word-retrieval compared against general fluid intelligence. This is crucial to understand whether tDCS responsiveness is linked to the functioning of the targeted language networks or to the functioning of the brain more broadly.

A further issue is the question of whether the timing of tDCS delivery (online vs. offline) influences its efficacy. Online stimulation, administered during task performance, enhances task-related neural activity, while offline stimulation, applied before the task, relies on the immediate changes in synaptic plasticity induced by stimulation [8, 12]. Age-related differences in neuroplasticity may affect the timing of tDCS effects, with younger adults often showing immediate benefits and older adults experiencing delayed responses [12]. Previous research investigating the use of tDCS to improve word retrieval is inconsistent regarding the timing of stimulation [43]. Clarifying the role of timing in tDCS effectiveness for young and older adults is crucial for optimizing protocols.

In addition to timing, the choice of target region may also modulate tDCS effects. Both the left IFG and left temporoparietal junction (TPJ) are integral to the naming network, but they may serve distinct roles. The left IFG is associated with controlled semantic retrieval and phonological processing, while the left TPJ is involved in integrating semantic and sensory information [16]. Previous studies suggest dissociable effects of stimulation to these regions, with left IFG stimulation improving action naming and left TPJ stimulation enhancing object naming [10, 25]. However, these effects have not been systematically explored in healthy aging populations. Understanding the differential impact of stimulation across language-related regions could help clarify previous inconsistencies and improve the design and efficacy of future clinical applications.

To address these gaps, the present study investigates the effects of focal tDCS on word retrieval in healthy young and older adults, focusing on two key language hubs in the naming network: the left IFG and the left TPJ. We assess the effects of online and offline stimulation and examine the moderating influence of baseline cognitive performance, on tDCS responsiveness. Our preregistered hypotheses (https://osf.io/wsmxt) predicted that: (1) tDCS would have a greater effect on older adults than younger adults, (2) stimulation of the left IFG would improve action naming, while stimulation of the left TPJ would improve object naming, (3) individuals with lower baseline cognitive performance in word retrieval would show the greatest benefit from tDCS. These results will provide valuable insights for future research and clinical applications of tDCS interventions in older adults.

## Methods

The study design and all analyses were preregistered online (https://osf.io/wsmxt).

### Participants

A power analysis was performed using G*Power [7]. In order to detect a medium effect size (η^2^ₚ = 0.06, f = 0.25), with an alpha level of 0.05, and a power of 0.80, 36 participants per condition were required, leading to a total sample size of 144 participants (36 younger left IFG, 36 younger left TPJ, 36 older left IFG, 36 older left TPJ).

Participants were neurologically healthy, right-handed, native English speakers who were tDCS-naive. Young adults were aged 18–30 years, and older adults were aged 55–85 years. Both young and older adults were stratified to receive either left IFG or left TPJ stimulation in a sham-controlled, repeated-measures design. The young IFG group (25 F/11M), young TPJ group (28 F/8M), older IFG group (22 F/14M), and older TPJ group (27 F/9M) were comparable in terms of gender distribution. All young adults were current undergraduate students. Older adults were recruited from the University of the Third Age (U3 A) and the community surrounding The University of Kent. The older adults were provided with £15 as compensation for their time. Young adults were recruited from the University of Kent and the surrounding community and received either course credits or £15 compensation for their time.

Standard tDCS safety guidelines for exclusions were followed [1], and included history of seizures or migraines, metallic objects in the head, electrical medical equipment on the person that was unable to be removed. None of the participants reported using medicines that are known to interfere with the effects of tDCS. Medical histories were provided by participants, and those diagnosed with neurological or mental disorders (such as dementia, major depression, stroke, or traumatic brain injury) were excluded. The study was conducted in compliance with the ethical standards outlined in the Declaration of Helsinki. All procedures involving human participants were reviewed and approved by the University of Kent Psychology Research Ethics Committee [ID: 202216587800677857], and informed consent was obtained from all participants prior to their participation.

### Picture naming task

A total of 200 black-and-white object drawings and 200 action drawings were selected from the International Picture Naming Project [2]. These stimuli were divided into four matched sets: two for object naming (one for online naming, one for offline naming) and two for action naming (one for online naming, one for offline naming). Each set was carefully balanced for age of acquisition, word frequency, visual complexity, name agreement percentage, syllable length, and character length, based on the extended norms and validation study by Székely et al. [40], ensuring comparable difficulty across conditions (see Supplementary Table 1).

Each trial began with a one-second fixation cross, followed by a bell sound, marking the start of response time measurement. The picture remained on the screen for three seconds or until a response was provided. Participants were instructed to name each picture as quickly and accurately as possible.

**Fig. 1:**
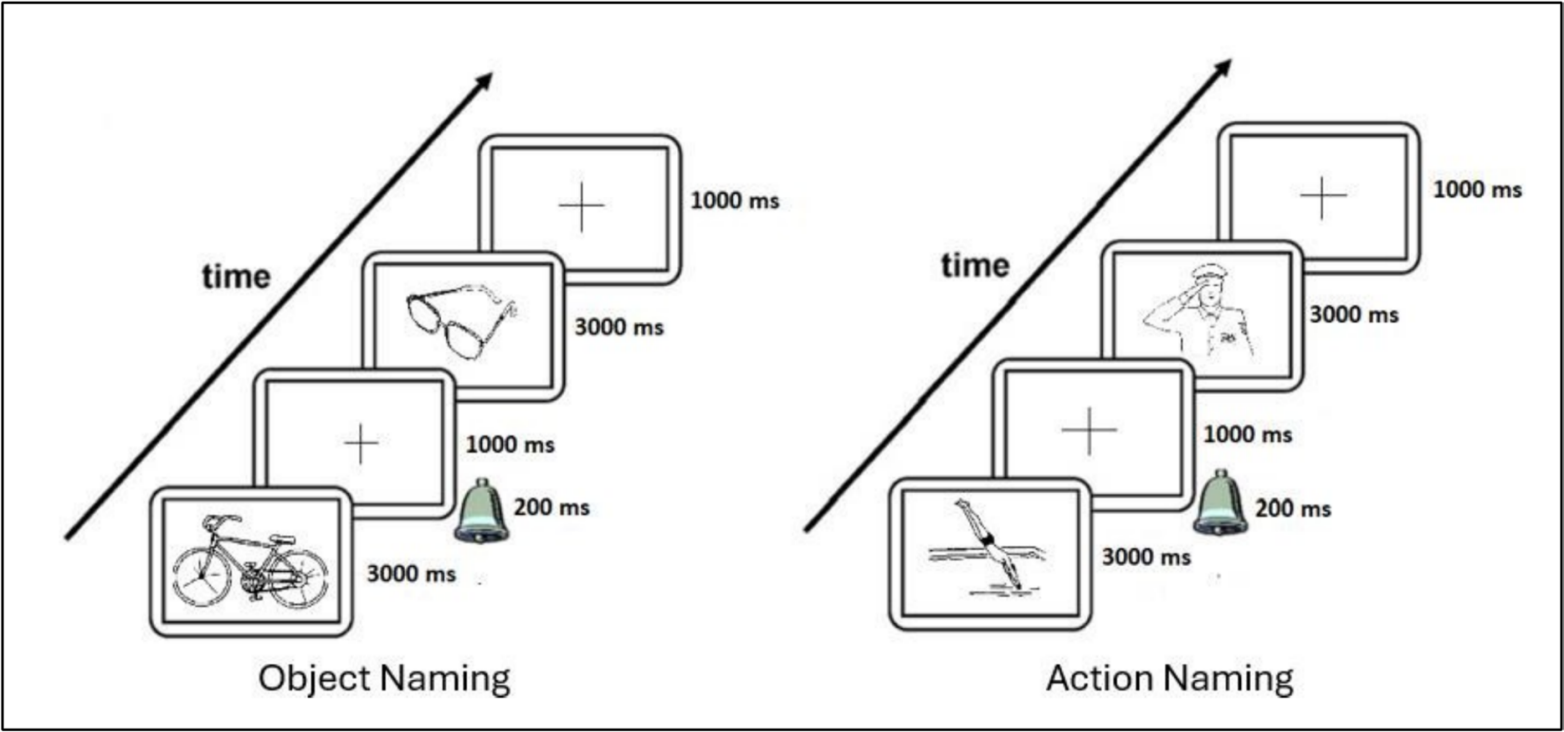
Picture naming task procedure

Object and action naming blocks were presented separately, with breaks offered between blocks. Object naming was always completed before action naming to maintain a consistent task sequence. Semantically related responses were accepted if deemed appropriate by a speech and language therapist (AY). Response times were recorded using the Black Box Toolkit (https://www.blackboxtoolkit.com/urpvk.html). Accuracy was recorded but not analyzed, as performance was near ceiling across participants. A schematic representation of the task procedure is provided in Fig. 1.

### Fluid intelligence

The Matrix Reasoning Item Bank (MaRs-IB; [5]) was used to evaluate fluid intelligence. Participants had to choose the right tile from four possibilities after being presented a 3 × 3 matrix of abstract patterns with one missing tile. An open-source substitute for conventional matrix reasoning tasks frequently employed in fluid intelligence assessments, like those found in standard IQ tests, is the MaRs-IB. These tasks show consistent age-related deterioration and are substantially associated with Spearman's g factor [26].

Before moving on to the test phase, which included 40 trials, participants finished five practice trials. Fluid intelligence was measured using accuracy scores, which were determined by adding together all of the correctly completed matrices.

### Baseline naming task

The baseline naming task was a shortened version of the main naming task, consisting of ten object and ten action images. These images differed from those used in the main task but were carefully matched for age of acquisition, word frequency, visual complexity, name agreement, syllable count, and character length. Average response time for correct responses was calculated and used to assess tDCS responsiveness in the analyses.

### Focal transcranial direct current stimulation

**Fig. 2:**
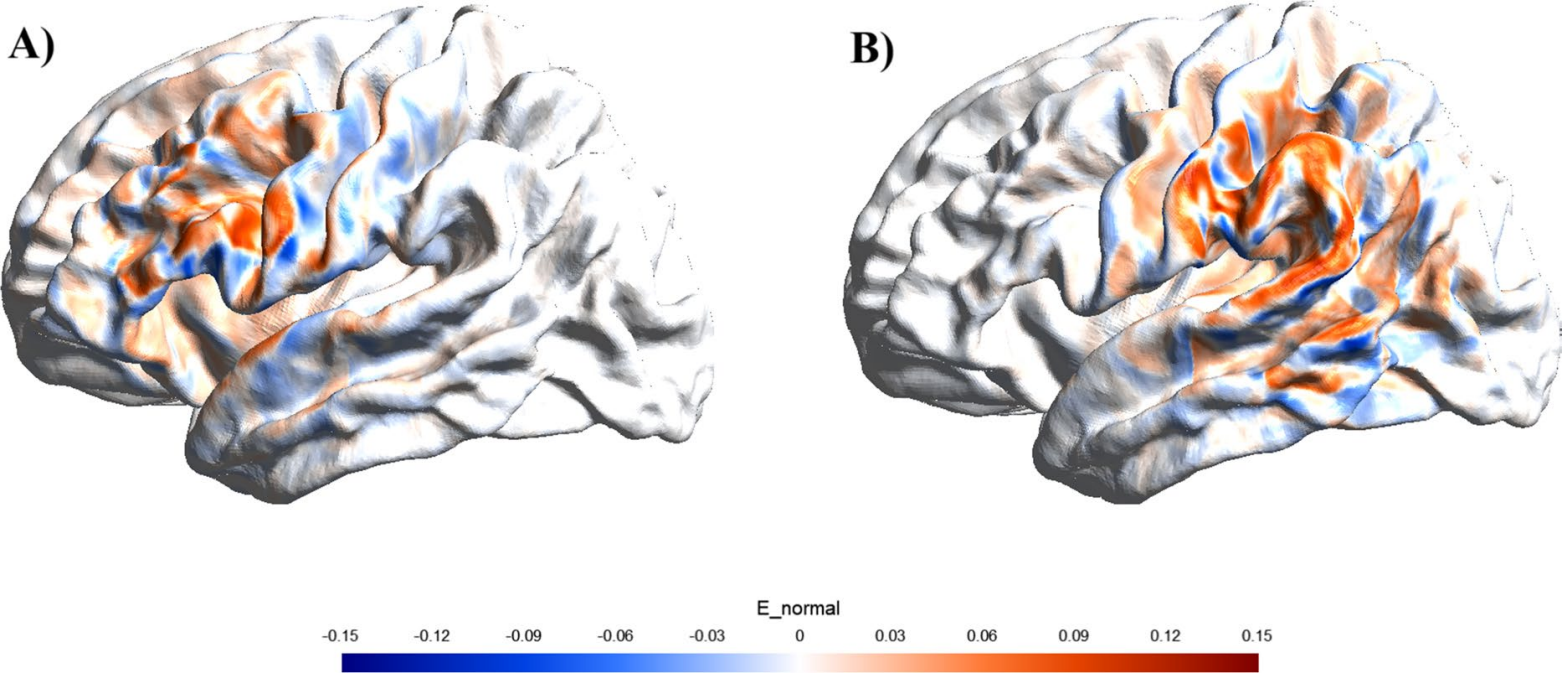
Surface rendering of the distribution and intensity of the estimated electric field for the two target sites with anodal stimulation. **A** Left inferior frontal gyrus, **B** Left temporoparietal junction. We present the normal component

We administered tDCS using a battery-driven, one-channel direct current stimulator (DC- Stimulator Plus, NeuroConn, Ilmenau, Germany, with double-blind, sham control option). The focal tDCS electrode montage comprised two concentric conductive rubber electrodes, a small circular centre electrode (diameter = 2.5 cm), and a ring-shaped return electrode (inner diameter = 7.5 cm; outer diameter = 9.8 cm). The centre electrode was positioned over the left inferior frontal gyrus (position FC5 of the 10–20 EEG system) or left temporoparietal junction (position CP5 of the 10–20 EEG system). Current modelling for both the left IFG and right TPJ is provided in Figure 2. The modelling of current flow was based on a realistic 1 mm MNI152 T1 standard brain of a young adult. Current modelling was performed using SimNIBS version 4.1. We present the normal component of the E-field (the component orthogonal to the cortical surface) considered the most physiologically relevant for modulating neuronal activity [35]. As no MRI data was available to run individualised modelling, the modelling presented in the current study should be considered as approximations of current flow based on a standard brain from a young adult.

The current was ramped up over 5 seconds to 1 mA during both stimulation conditions. In the anodal condition the current remained at 1 mA for 20 mins before ramping down over 5 seconds. In the sham condition, the current ramped up to 1 mA over 5 seconds and was ramped down after 40 seconds. The sham condition generates a tactile sensation comparable to the active stimulation. This approach has been shown to effectively blind subjects as to which stimulation they have received [14, 27, 29, 28]. We used the"study mode"of the DC stimulator, which involves a predefined code that triggers either active or sham tDCS, to achieve investigator blinding. The codes were assigned by a researcher who did not participate in data collection.

### Mood, adverse effects, and blinding

Participant’s mood was evaluated using the Visual Analogue Mood Scale (VAMS; [11]) before and after each session. The VAMS assessed current positive and negative emotional states on visual analogue scales ranging from 0 to 100 (i.e., afraid, confused, sad, angry, energetic, tired, happy, and tense). Higher scores indicate greater intensity. Mood change scores were calculated by subtracting pre-stimulation scores from the post-stimulation scores for each mood. We calculated two mood change scores; positive and negative mood by summing the respective mood change scores.

Adverse effects were assessed using the self-report questionnaire developed by Brunoni et al. [3]. Participants graded the severity (1 = absent, 2 = mild, 3 = moderate, 4 = severe) and incidence of a variety of potential adverse effects, which consisted of headache, neck pain, scalp pain, tingling, itching, burning sensation, skin redness, sleepiness, trouble concentrating, and acute mood changes. Participant blinding was assessed at the completion of the study. Participants were asked, “Do you think the active stimulation was in the first or second session?” Participants were forced to answer session one or two, even when they were unsure.

### Procedure

**Fig. 3:**
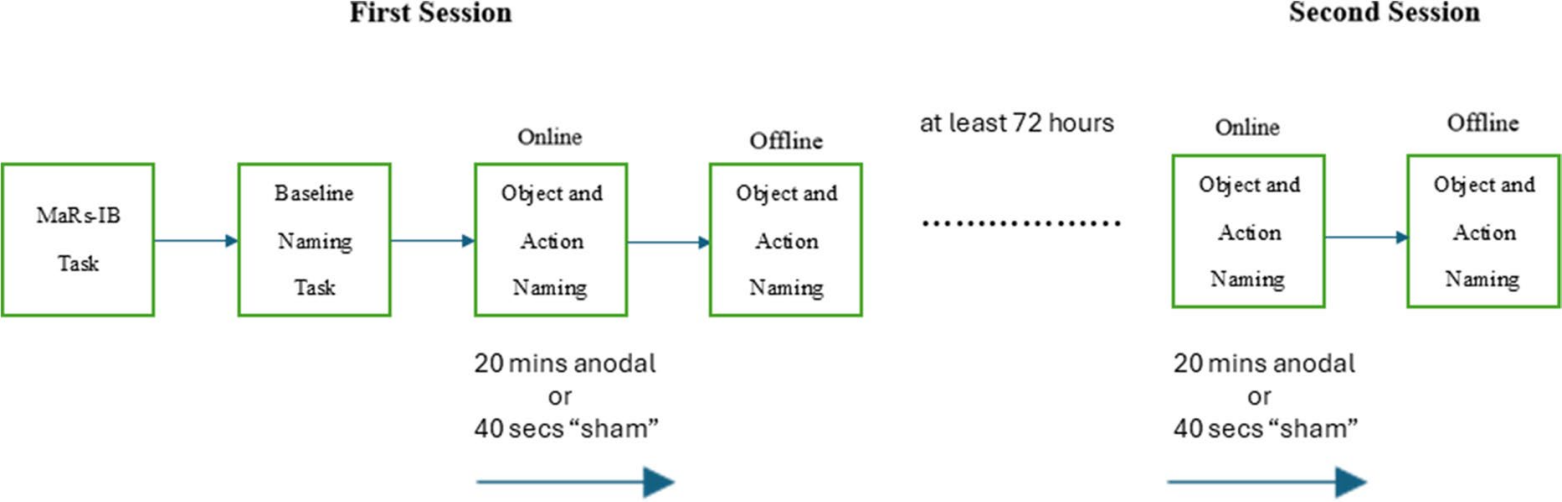
Schematic detailing the procedure across the two stimulation sessions

The study followed a double-blind, sham-controlled, repeated-measures tDCS design and was conducted in dedicated brain stimulation laboratories within The School of Psychology at the University of Kent. Participants were stratified to receive either left IFG or left TPJ stimulation. Participation was conducted over two experimental sessions, one with anodal stimulation and one with sham stimulation, which were at least 72 h apart to avoid carryover effects with order counterbalanced. Before the first session, participants completed the fluid intelligence assessment, followed by the baseline object and action naming task, and the pre-stimulation mood scale. Stimulation started just prior to participants starting the picture-naming task (online condition). After stimulation ended, participants completed a second version of the picture-naming task without any active stimulation (offline condition). Participants then completed the post-stimulation mood scale and the adverse effects checklist. The outline is presented in Fig. 3.

### Statistical analyses

We conducted a 2 × 2× 2× 2× 2 repeated-measures ANOVA to assess whether online or offline stimulation (Stimulation Time) to the left IFG or left TPJ (Region) modified object or action naming (Naming Type) in healthy younger and older adults (Age Group) using a sham-controlled design (anodal v sham; Stimulation Type). All factors were within-subject, except Age Group and Brain Region, which were between-subject. The dependent variable was response speed in milliseconds (ms).

Where null effects were identified for hypothesized effects, Bayesian analyses were conducted using JASP 0.18.3 [18], to assess the confidence in the null effect.

In follow-up analyses, we explored whether baseline naming ability predicted stimulation response in older adults. A 2 × 2× 2× 2 repeated-measures ANCOVA was conducted with baseline naming and fluid intelligence as covariates. The same analysis was also conducted in younger adults.

We assessed blinding across each age group and stimulation site using chi-square tests. Mood change was assessed by calculating the difference on a combined positive scale (happy and energetic) and combined negative scales (tired, afraid, sad, tense, confused, angry) of the VAMS before and after both anodal and sham stimulation. The positive and negative mood change scores were the dependent variables in 2 × 2× 2 ANOVAs, with age group (young vs. older), stimulation site (left IFG vs. left TPJ), and stimulation type (sham vs. anodal) as independent variables.

## Results

The groups were comparable in terms of gender distribution, χ^2^(3, *N* = 171) = 2.82, *p* = 0.42. The mean age of older adults did not differ between the left IFG (M = 71.8 years, SD = 6.1) and left TPJ groups (M = 71.4 years, SD = 7.2), t(70) = 0.25, *p* = 0.81, d = 0.06. Among young adults, those in the left IFG group (M = 19.6 years, SD = 2.2) were slightly older than those in the left TPJ group (M = 18.7 years, SD = 1.2), t(70) = 2.29, *p* =.02, d = 0.24.

The older groups were also comparable in terms of education level (data missing for three participants). Education level did not significantly differ between the left IFG group (M = 15.9 years, SD = 2.0) and the left TPJ group (M = 15.3 years, SD = 2.4), t(67) = 1.06, *p* =.30, d = 0.25. All young adults were undergraduate students. Descriptive statistics of performance (response times in milliseconds) on object and action naming during the online and offline phases of the stimulation across the stimulation site, age group, and stimulation type are provided in Table 1.

**Table 1:** Descriptive statistics of performance (response times in milliseconds) on object and action naming during the online and offline phases of the stimulation across the stimulation site, age group, and stimulation type

	Left inferior frontal gyrus				Left temporoparietal junction			
	Older adults		Younger adults		Older adults		Younger adults	
	Sham	Anodal	Sham	Anodal	Sham	Anodal	Sham	Anodal
	Mean (sd)	Mean (sd)	Mean (sd)	Mean (sd)	Mean (sd)	Mean (sd)	Mean (sd)	Mean (sd)
Object online	1053 (219)	1038 (195)	1060 (187)	1057 (177)	1011 (140)	1004 (142)	1038 (191)	1033 (208)
Object offline	1023 (206)	1007 (170)	974 (185)	1011 (172)	1009 (187)	980 (158)	992 (198)	1011 (220)
Action online	1331 (212)	1301 (197)	1368 (207)	1344 (208)	1281 (185)	1261 (199)	1326 (196)	1346 (236)
Action offline	1314 (200)	1268 (176)	1420 (199)	1441 (216)	1301 (191)	1309 (206)	1396 (205)	1402 (216)

A main effect of Naming Type was identified, *F*(1, 140) = 1607.87, *p* <.001, η^2^ₚ = 0.92, but this was subsumed within a significant Age Group × Naming Type interaction, *F*(1, 140) = 24.31, *p* <.001, η^2^ₚ = 0.15. The post hoc analysis identified slower action naming in younger adults (*M* = 1380 ms) than in older adults (*M* = 1294 ms). No difference was identified for object naming in younger adults (*M* = 1022 ms) and older adults (*M* = 1014 ms).

The main effect of Stimulation was not significant, *F*(1, 140) = 0.37, *p* = 0.54, η^2^ₚ = 0.003. Likewise, stimulation had no effect on performance dependent upon Region, Age Group, Stimulation Time, or Naming Type, nor any interaction between these variables (*p*s between 0.09 and 0.99) Several effects were identified that were not central to the present study; see Supplementary Table 2 for full details). At the left IFG, Bayesian analyses returned moderate evidence for the null hypothesis for the main effect of stimulation (BF_10_ = 0.16), with weak evidence for the interactions between stimulation x naming type (BF_10_ = 0.74), stimulation x age group (BF_10_ = 0.53), and stimulation x naming type x age group (BF_10_ = 0.34). At the left TPJ, the evidence for the null hypothesis for an overall stimulation effect was moderate (BF_10_ = 0.19). Likewise for the interaction between stimulation x naming type (BF_10_ = 0.23). The evidence was weak for the interaction between stimulation x age group (BF_10_ = 0.34) and moderate for the interaction between stimulation x naming type x age group (BF_10_ = 0.25).

Therefore, our hypotheses pertaining to stimulation effects at both the left IFG and left TPJ were not supported.

### Stimulation effects dependent on baseline functioning

The interaction between Baseline Naming × Stimulation Type × Region was significant, *F*(1, 66) = 4.41, *p* =.04, η^2^ₚ = 0.06. However, the Fluid Intelligence × Stimulation Type × Region interaction was not significant, *F*(1, 66) = 0.38, *p* =.54, η^2^ₚ = 0.01. Further interactions, including Baseline Naming × Stimulation Type × Region × Stimulation Time, *F*(1, 68) = 0.34, *p* =.56, η^2^ₚ = 0.001, and Baseline Naming × Stimulation Type × Region × Stimulation Time × Naming Type, *F*(1, 68) = 0.08, *p* =.77, η^2^ₚ = 0.001, were not significant. The full statistical model is provided in Supplementary 3.

**Fig. 4:**
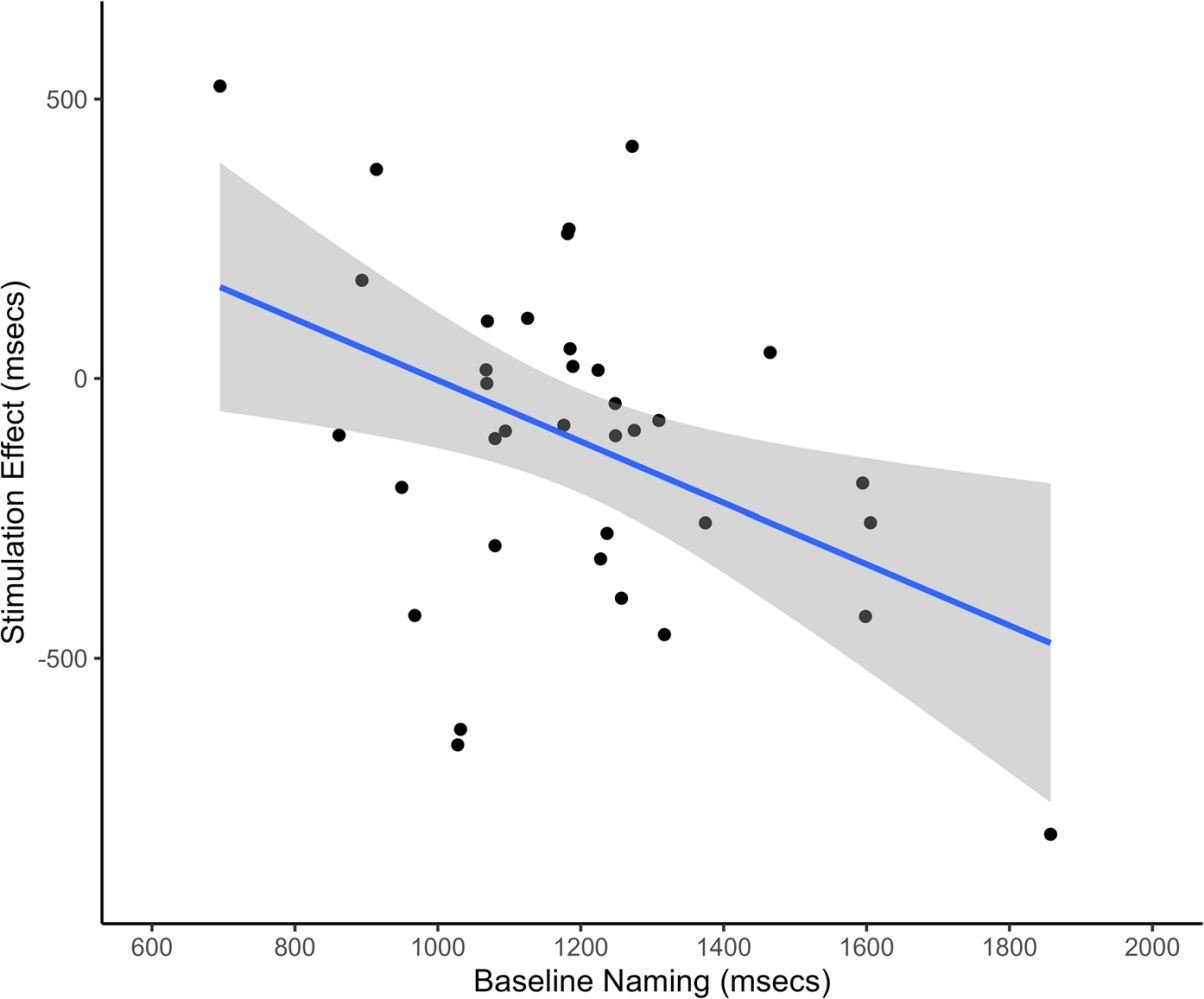
Stimulation effect at the left inferior frontal gyrus was dependent on baseline naming speed

To explore the significant interaction, we conducted separate analyses for each brain region. For the left IFG, the Baseline Naming × Stimulation Type interaction was significant, *F*(1, 33) = 6.74, *p* =.01 (*p*=.02 Bonferroni corrected), η^2^ₚ = 0.17. For the left TPJ, this interaction was not significant, *F*(1, 33) = 0.65, *p* =.43 (*p* =.86 Bonferroni corrected), η^2^ₚ = 0.02. Scatterplots (see Figure 4) showed that the stimulation effect (calculated as the difference in response speed for active and sham stimulation) was negatively correlated with baseline naming performance. Therefore, in older adults, stimulation to the left IFG improved naming speed in those who performed slower during the baseline assessment.

We conducted the same analysis in younger adults, but no relationship was identified for baseline naming or fluid intelligence on any of the stimulation effects (*p*s between.11 and.91; see Supplementary Table 3 for more details).

Although there were no overall stimulation effects, we provide evidence that baseline naming ability moderated tDCS responsiveness at the left IFG.

### Blinding, adverse effects, and mood change

Blinding was achieved in older adults, although younger adults were better than chance at predicting the active stimulation. A slight reduction in positive mood was experienced after active stimulation to either brain region, but no effects were identified for negative mood. Adverse effects were comparable across sham and active sessions. See Supplementary 4 for full analyses and results.

## Discussion

We investigated the effect of online and offline focal stimulation to either the left inferior frontal gyrus (left IFG) or left temporoparietal junction (left TPJ) on object and action naming in young and older adults. Additionally, we assessed whether baseline cognitive performance predicted stimulation response in older adults. Our findings indicated that focal stimulation to the left IFG, but not the left TPJ, was associated with improved naming in older adults with lower baseline performance. However, no overall effect of stimulation was observed across the entire sample, highlighting the importance of considering individual differences in response to tDCS.

These findings align with previous research suggesting that tDCS effects may be most evident in individuals with lower cognitive performance [22, 32, 34]. The inconsistent effects reported in prior literature [20] may reflect variability in baseline performance and requires consideration in future tDCS studies in healthy older adults. Importantly, our study did not find evidence of differential effects between online and offline stimulation, but did find site-specific effects at the left IFG. The results were not explained by unblinding as older adults were unable to correctly identify active versus sham stimulation above chance level. It is interesting to note that younger adults showed greater accuracy in identifying stimulation conditions. This pattern is consistent with prior studies [13, 19] and may be attributed to age-related differences in skin conductance and sensory perception.

Our results provide partial support for previous studies indicating site-specific effects of tDCS on language processing. However, the lack of a main effect of stimulation, coupled with small effect sizes, suggests that the clinical utility of tDCS for language enhancement in healthy ageing remains uncertain. Previous studies have reported that stimulation to the left IFG facilitates action naming, while stimulation to the left TPJ enhances object naming [9, 10, 25]. In contrast, our findings did not reveal such a dissociation, instead indicating that left IFG stimulation was associated with faster naming of both object and action words and only in those that require a *boost*. This discrepancy may stem from differences in sample size and task design, as previous studies often employed smaller cohorts and fewer stimuli. The results from the present study highlight the difficulty of improving cognition in healthy older adults and in replicating previous tDCS effects.

While these findings offer preliminary evidence for baseline-dependent effects, it is important to note that replication is required to increase confidence in this effect, optimally in much larger samples better suited to identifying correlations between baseline functioning and tDCS responsiveness. The absence of robust group-wide effects suggests that tDCS may not be a consistently effective tool for enhancing language function in healthy older adults. Instead, our results contribute to a growing body of literature highlighting the variability and difficulty in replicating tDCS effects. One consideration for future research is to provide a more personalised approach to stimulation. Prior studies using conventional tDCS may have inadvertently stimulated adjacent regions, such as the motor cortex or superior temporal gyrus [36], and although this issue is reduced when using focal stimulation, electrode placement variability can have a greater influence on underlying neural activation [33]. Future research should employ more precise stimulation techniques, such as neuronavigation-guided tDCS.

Although our study provides insights into the timing and site-specific effects of focal stimulation, several limitations warrant discussion. The use of EEG-based electrode placement did not account for individual anatomical variability, potentially contributing to inconsistent effects. Future studies should employ individualized structural imaging to optimize electrode positioning and better understand interindividual differences in current flow [37]. It should also be noted that our current estimations are based on a standard young brain template. Age-related changes to both brain structure and function have been shown to affect current flow [24] and should be considered in future research. Third, the short-term nature of our study precludes conclusions regarding the long-term efficacy of tDCS for language function. Prior research suggests that multisession tDCS may yield more sustained cognitive benefits [34], warranting further investigation into the duration and generalizability of naming improvements. Additionally, our sample comprised healthy older adults, limiting the applicability of our findings to clinical populations with language impairments such as aphasia, semantic dementia, or Parkinson's disease [21, 41, 44]. Although our sample is large for tDCS, larger samples may be required to confidently associate baseline performance with tDCS responsiveness. The left IFG is often targeted via FC5 of the 10–20 EEG system and this resulted in excitation predominantly in the dorsal portion of the left IFG and middle frontal gyrus. Future research could target more ventral regions of the left IFG.

In conclusion, our study demonstrates that focal tDCS to the left IFG may improve naming speed in older adults with lower baseline performance. However, the absence of an overall effect of stimulation, coupled with weak effect sizes, underscores the challenges in replicating tDCS effects and questions its broad applicability as a language enhancement tool. Future research should explore multisession protocols, individualized electrode targeting, and clinical applications to further elucidate the potential of tDCS for language rehabilitation.

## Supplementary Information

Below is the link to the electronic supplementary material.

Supplementary file1 (DOCX 19 KB)

Supplementary file2 (DOCX 34 KB)

Supplementary file3 (DOCX 39 KB)

Supplementary file4 (DOCX 27 KB)

## Data Availability

Data will be available online at (https://osf.io/wsmxt).
