# Supplementary material for: Regionally specific picture naming benefits of focal tDCS are dependent on baseline performance in older adults
Source: GeroScience. 2025 May 10;47(6):6839–49. doi: 10.1007/s11357-025-01674-x (PMC12638570; doi:10.1007/s11357-025-01674-x)
Supplement: Supplementary file 1 — Supplementary file1 (DOCX 19 KB) [file 11357_2025_1674_MOESM1_ESM.docx]

**Supplementary Table 1.** Linguistic and visual factors for Object and Action Picture Lists

| Object Pictures | List 1 | List 2 | Baseline | ANOVA p-value |
| --- | --- | --- | --- | --- |
|  | Mean (sd) | Mean (sd) | Mean (sd) |  |
| Age of Acquisition | 2.320 (0.891) | 2.200 (0.904) | 2.600 (0.843) | .416 |
| Name Agreement | 0.851 (0.179) | 0.856 (0.161) | 0.865 (0.146) | .971 |
| Syllable Length | 1.720 (0.701) | 1.860 (1.050) | 2.400 (0.843) | .091 |
| Character Length | 5.900 (2.092) | 5.900 (2.589) | 6.900 (2.183) | .439 |
| Frequency | 2.353 (1.674) | 2.344 (1.625) | 2.215 (1.740) | .971 |
| Visual Complexity | 18597 (12663) | 16144 (6964) | 20277 (12119) | .353 |
| Action Pictures |  |  |  |  |
|  | Mean (sd) | Mean (sd) | Mean (sd) |  |
| Age of Acquisition | 2.500 (0.735) | 2.660 (0.658) | 2.100 (0.994) | .08 |
| Name Agreement | 0.707 (0.242) | 0.723 (0.228) | 0.653 (0.238) | .687 |
| Syllable Length | 1.200 (0.452) | 1.180 (0.388) | 1.300 (0.675) | .742 |
| Character Length | 4.740 (1.337) | 4.400 (1.010) | 4.400 (1.350) | .338 |
| Frequency | 3.435 (1.335) | 3.515 (1.698) | 4.092 (1.595) | .465 |
| Visual Complexity | 21531 (6776) | 21708 (6636) | 21926 (8011) | .983 |
