# Supplementary material for: Regionally specific picture naming benefits of focal tDCS are dependent on baseline performance in older adults
Source: GeroScience. 2025 May 10;47(6):6839–49. doi: 10.1007/s11357-025-01674-x (PMC12638570; doi:10.1007/s11357-025-01674-x)
Supplement: Supplementary file 2 — Supplementary file2 (DOCX 34 KB) [file 11357_2025_1674_MOESM2_ESM.docx]

**Supplementary Table 2. Effects of stimulation to the lIFG and lTPJ in young and older adults**

| **Within Subjects Effects** | | | | | | | | | | | | | | |  |
| --- | --- | --- | --- | --- | --- | --- | --- | --- | --- | --- | --- | --- | --- | --- | --- |
| **Cases** | **Sum of Squares** | | | **df** | | **Mean Square** | | | **F** | | **p** | | **η²_p_** | |  |
| Stimulation Type |  | 0.008 |  | | 1 |  | 0.008 |  | | 0.369 |  | 0.545 |  | 0.003 |  |
| Stimulation Type ✻ Region |  | 0.005 |  | | 1 |  | 0.005 |  | | 0.242 |  | 0.624 |  | 0.002 |  |
| Stimulation Type ✻ Age Group |  | 0.056 |  | | 1 |  | 0.056 |  | | 2.766 |  | 0.099 |  | 0.019 |  |
| Stimulation Type ✻ Region ✻ Age Group |  | 0.002 |  | | 1 |  | 0.002 |  | | 0.121 |  | 0.728 |  | <0.001 |  |
| Residuals |  | 2.846 |  | | 140 |  | 0.020 |  | |  |  |  |  |  |  |
| Naming Type |  | 29.351 |  | | 1 |  | 29.351 |  | | 1607.871 |  | < .001 |  | 0.920 |  |
| Naming Type ✻ Region |  | <0.001 |  | | 1 |  | <0.001 |  | | 0.024 |  | 0.877 |  | <0.001 |  |
| Naming Type ✻ Age Group |  | 0.444 |  | | 1 |  | 0.444 |  | | 24.306 |  | < .001 |  | 0.148 |  |
| Naming Type ✻ Region ✻ Age Group |  | 0.019 |  | | 1 |  | 0.019 |  | | 1.066 |  | 0.304 |  | 0.008 |  |
| Residuals |  | 2.556 |  | | 140 |  | 0.018 |  | |  |  |  |  |  |  |
| Stimulation Time |  | <0.001 |  | | 1 |  | <0.001 |  | | 0.003 |  | 0.954 |  | <0.001 |  |
| Stimulation Time ✻ Region |  | 0.042 |  | | 1 |  | 0.042 |  | | 4.420 |  | 0.037 |  | 0.031 |  |
| Stimulation Time ✻ Age Group |  | 0.023 |  | | 1 |  | 0.023 |  | | 2.398 |  | 0.124 |  | 0.017 |  |
| Stimulation Time ✻ Region ✻ Age Group |  | 0.014 |  | | 1 |  | 0.014 |  | | 1.466 |  | 0.228 |  | 0.010 |  |
| Residuals |  | 1.341 |  | | 140 |  | 0.010 |  | |  |  |  |  |  |  |
| Stimulation Type ✻ Naming Type |  | 0.002 |  | | 1 |  | 0.002 |  | | 0.238 |  | 0.626 |  | 0.002 |  |
| Stimulation Type ✻ Naming Type ✻ Region |  | 0.015 |  | | 1 |  | 0.015 |  | | 1.677 |  | 0.197 |  | 0.012 |  |
| Stimulation Type ✻ Naming Type ✻ Age Group |  | <0.001 |  | | 1 |  | <0.001 |  | | 0.005 |  | 0.946 |  | <0.001 |  |
| Stimulation Type ✻ Naming Type ✻ Region ✻ Age Group |  | <0.001 |  | | 1 |  | <0.001 |  | | 0.063 |  | 0.802 |  | <0.001 |  |
| Residuals |  | 1.277 |  | | 140 |  | 0.009 |  | |  |  |  |  |  |  |
| Stimulation Type ✻ Stimulation Time |  | 0.008 |  | | 1 |  | 0.008 |  | | 0.958 |  | 0.329 |  | 0.007 |  |
| Stimulation Type ✻ Stimulation Time ✻ Region |  | 0.003 |  | | 1 |  | 0.003 |  | | 0.362 |  | 0.548 |  | 0.003 |  |
| Stimulation Type ✻ Stimulation Time ✻ Age Group |  | 0.013 |  | | 1 |  | 0.013 |  | | 1.666 |  | 0.199 |  | 0.012 |  |
| Stimulation Type ✻ Stimulation Time ✻ Region ✻ Age Group |  | 0.011 |  | | 1 |  | 0.011 |  | | 1.406 |  | 0.238 |  | 0.010 |  |
| Residuals |  | 1.113 |  | | 140 |  | 0.008 |  | |  |  |  |  |  |  |
| Naming Type ✻ Stimulation Time |  | 0.378 |  | | 1 |  | 0.378 |  | | 70.427 |  | < .001 |  | 0.335 |  |
| Naming Type ✻ Stimulation Time ✻ Region |  | <0.001 |  | | 1 |  | <0.001 |  | | 0.011 |  | 0.918 |  | <0.001 |  |
| Naming Type ✻ Stimulation Time ✻ Age Group |  | 0.153 |  | | 1 |  | 0.153 |  | | 28.456 |  | < .001 |  | 0.169 |  |
| Naming Type ✻ Stimulation Time ✻ Region ✻ Age Group |  | 0.033 |  | | 1 |  | 0.033 |  | | 6.150 |  | 0.014 |  | 0.042 |  |
| Residuals |  | 0.751 |  | | 140 |  | 0.005 |  | |  |  |  |  |  |  |
| Stimulation Type ✻ Naming Type ✻ Stimulation Time |  | <0.001 |  | | 1 |  | <0.001 |  | | <0.001 |  | 0.987 |  | <0.001 |  |
| Stimulation Type ✻ Naming Type ✻ Stimulation Time ✻ Region |  | <0.001 |  | | 1 |  | <0.001 |  | | 0.065 |  | 0.799 |  | <0.001 |  |
| Stimulation Type ✻ Naming Type ✻ Stimulation Time ✻ Age Group |  | 0.005 |  | | 1 |  | 0.005 |  | | 0.561 |  | 0.455 |  | 0.004 |  |
| Stimulation Type ✻ Naming Type ✻ Stimulation Time ✻ Region ✻ Age Group |  | 0.013 |  | | 1 |  | 0.013 |  | | 1.494 |  | 0.224 |  | 0.011 |  |
| Residuals |  | 1.244 |  | | 140 |  | 0.009 |  | |  |  |  |  |  |  |
|  | | | | | | | | | | | | | | |  |
| *Note.*  Type III Sum of Squares | | | | | | | | | | | | | | |  |

| **Between Subjects Effects** | | | | | | | | | | | | | |
| --- | --- | --- | --- | --- | --- | --- | --- | --- | --- | --- | --- | --- | --- |
| **Cases** | | **Sum of Squares** | | **df** | | **Mean Square** | | **F** | | **p** | | **η²_p_** | |
| Region |  | 0.110 |  | 1 |  | 0.110 |  | 0.490 |  | 0.485 |  | 0.003 |  |
| Age Group |  | 0.590 |  | 1 |  | 0.590 |  | 2.616 |  | 0.108 |  | 0.018 |  |
| Region ✻ Age Group |  | 0.003 |  | 1 |  | 0.003 |  | 0.014 |  | 0.908 |  | 9.667×10^-5^ |  |
| Residuals |  | 31.570 |  | 140 |  | 0.226 |  |  |  |  |  |  |  |
|  | | | | | | | | | | | | | |
| *Note.*  Type III Sum of Squares | | | | | | | | | | | | | |

**Descriptives**

| **Stimulation Type** | **Naming Type** | **Stimulation Time** | **Region** | **N** | **Mean** | **SD** | **SE** |
| --- | --- | --- | --- | --- | --- | --- | --- |
| Sham | Object | Online | F | 36 | 1053 | 219 | 37 |
|  |  |  | T | 36 | 1011 | 140 | 23 |
|  |  | Offline | F | 36 | 1023 | 206 | 34 |
|  |  |  | T | 36 | 1009 | 187 | 31 |
|  | Action | Online | F | 36 | 1331 | 212 | 35 |
|  |  |  | T | 36 | 1281 | 185 | 31 |
|  |  | Offline | F | 36 | 1314 | 200 | 33 |
|  |  |  | T | 36 | 1301 | 191 | 32 |
| Anodal | Object | Online | F | 36 | 1038 | 195 | 33 |
|  |  |  | T | 36 | 1004 | 142 | 24 |
|  |  | Offline | F | 36 | 1007 | 170 | 28 |
|  |  |  | T | 36 | 980 | 158 | 26 |
|  | Action | Online | F | 36 | 1301 | 197 | 33 |
|  |  |  | T | 36 | 1261 | 199 | 33 |
|  |  | Offline | F | 36 | 1268 | 176 | 29 |
|  |  |  | T | 36 | 1309 | 206 | 34 |

Supplementary Results

We show that participants generally were faster in the offline condition (M=1001ms) than the online condition (M=1037ms) for object naming, but were slower in the offline condition for action naming (M=1356ms) compared with the online condition (M=1320ms). However, this was subsumed under an interaction with age group and brain region. To explore this interaction we looked at the interaction between naming type and stimulation time in both young and older adults at both the left IFG and the left TPJ. There was no difference in naming x stimulation time x AGE GROUP in the left TPJ group, F(1,70)=3.64, *p*= .06, η²ₚ = 0.001, but the interaction was significant in the left IFG group, F(1,70)=34.64, *p*< .001, η²ₚ = 0.004. In the young adults, naming x stimulation time interaction was significant, F(1,35)=60.96, *p*< .001, η²ₚ = 0.03. In the older adults, naming x stimulation time interaction was not significant, F(1,35)=0.17 , *p*= 0.68, η²ₚ < 0.001. Younger adults in the left IFG group were slower for object naming in the online session (M=1059ms) compared to the offline session (M=992ms), but faster for action naming (M=1356ms) in the online session compared to the offline session (M=1431ms).
