# Supplementary material for: Regionally specific picture naming benefits of focal tDCS are dependent on baseline performance in older adults
Source: GeroScience. 2025 May 10;47(6):6839–49. doi: 10.1007/s11357-025-01674-x (PMC12638570; doi:10.1007/s11357-025-01674-x)
Supplement: Supplementary file 3 — Supplementary file3 (DOCX 39 KB) [file 11357_2025_1674_MOESM3_ESM.docx]

**Supplementary Table 3. Effects of baseline naming and fluid intelligence on stimulation effects in older adults**

| **Cases** | **Sum of Squares** | **df** | **Mean Square** | **F** | **p** | **η²p** |
| --- | --- | --- | --- | --- | --- | --- |
| Stimulation Type | < .001 | 1 | < .001 | 0.001 | 0.977 | < .001 |
| Stimulation Type ✻ Region | 0.014 | 1 | 0.014 | 1.111 | 0.296 | 0.017 |
| Stimulation Type ✻ Fluid Intelligence | 0.003 | 1 | 0.003 | 0.206 | 0.651 | 0.003 |
| Stimulation Type ✻ Baseline Naming Speed | 0.006 | 1 | 0.006 | 0.472 | 0.494 | 0.007 |
| Stimulation Type ✻ Region ✻ Baseline Naming Speed | 0.054 | 1 | 0.054 | 4.406 | 0.040 | 0.063 |
| Stimulation Type ✻ Region ✻ Fluid Intelligence | 0.005 | 1 | 0.005 | 0.378 | 0.541 | 0.006 |
| Residuals | 0.807 | 66 | 0.012 |  |  |  |
| Naming Type | 0.022 | 1 | 0.022 | 1.178 | 0.282 | 0.018 |
| Naming Type ✻ Region | 0.046 | 1 | 0.046 | 2.387 | 0.127 | 0.035 |
| Naming Type ✻ Fluid Intelligence | 0.003 | 1 | 0.003 | 0.172 | 0.680 | 0.003 |
| Naming Type ✻ Baseline Naming Speed | 0.087 | 1 | 0.087 | 4.557 | 0.037 | 0.065 |
| Naming Type ✻ Region ✻ Baseline Naming Speed | 0.089 | 1 | 0.089 | 4.674 | 0.034 | 0.066 |
| Naming Type ✻ Region ✻ Fluid Intelligence | < .001 | 1 | < .001 | 0.007 | 0.936 | < .001 |
| Residuals | 1.258 | 66 | 0.019 |  |  |  |
| Stimulation Time | 0.002 | 1 | 0.002 | 0.169 | 0.682 | 0.003 |
| Stimulation Time ✻ Region | 0.011 | 1 | 0.011 | 1.130 | 0.292 | 0.017 |
| Stimulation Time ✻ Fluid Intelligence | < .001 | 1 | < .001 | 0.045 | 0.834 | < .001 |
| Stimulation Time ✻ Baseline Naming Speed | 0.008 | 1 | 0.008 | 0.819 | 0.369 | 0.012 |
| Stimulation Time ✻ Region ✻ Baseline Naming Speed | < .001 | 1 | < .001 | 0.033 | 0.856 | < .001 |
| Stimulation Time ✻ Region ✻ Fluid Intelligence | 0.024 | 1 | 0.024 | 2.469 | 0.121 | 0.036 |
| Residuals | 0.640 | 66 | 0.010 |  |  |  |
| Stimulation Type ✻ Naming Type | 0.001 | 1 | 0.001 | 0.141 | 0.709 | 0.002 |
| Stimulation Type ✻ Naming Type ✻ Region | 0.010 | 1 | 0.010 | 1.229 | 0.272 | 0.018 |
| Stimulation Type ✻ Naming Type ✻ Fluid Intelligence | 0.007 | 1 | 0.007 | 0.875 | 0.353 | 0.013 |
| Stimulation Type ✻ Naming Type ✻ Baseline Naming Speed | < .001 | 1 | < .001 | 0.038 | 0.846 | < .001 |
| Stimulation Type ✻ Naming Type ✻ Region ✻ Baseline Naming Speed | 0.003 | 1 | 0.003 | 0.342 | 0.561 | 0.005 |
| Stimulation Type ✻ Naming Type ✻ Region ✻ Fluid Intelligence | 0.007 | 1 | 0.007 | 0.886 | 0.350 | 0.013 |
| Residuals | 0.539 | 66 | 0.008 |  |  |  |
| Stimulation Type ✻ Stimulation Time | 0.003 | 1 | 0.003 | 0.411 | 0.523 | 0.006 |
| Stimulation Type ✻ Stimulation Time ✻ Region | 0.002 | 1 | 0.002 | 0.254 | 0.616 | 0.004 |
| Stimulation Type ✻ Stimulation Time ✻ Fluid Intelligence | < .001 | 1 | < .001 | 0.092 | 0.763 | < .001 |
| Stimulation Type ✻ Stimulation Time ✻ Baseline Naming Speed | 0.003 | 1 | 0.003 | 0.365 | 0.548 | 0.006 |
| Stimulation Type ✻ Stimulation Time ✻ Region ✻ Baseline Naming Speed | 0.003 | 1 | 0.003 | 0.383 | 0.538 | 0.006 |
| Stimulation Type ✻ Stimulation Time ✻ Region ✻ Fluid Intelligence | < .001 | 1 | < .001 | 0.031 | 0.861 | < .001 |
| Residuals | 0.540 | 66 | 0.008 |  |  |  |
| Naming Type ✻ Stimulation Time | 0.003 | 1 | 0.003 | 0.650 | 0.423 | 0.010 |
| Naming Type ✻ Stimulation Time ✻ Region | < .001 | 1 | < .001 | 0.056 | 0.814 | < .001 |
| Naming Type ✻ Stimulation Time ✻ Fluid Intelligence | 0.010 | 1 | 0.010 | 2.035 | 0.158 | 0.030 |
| Naming Type ✻ Stimulation Time ✻ Baseline Naming Speed | < .001 | 1 | < .001 | 0.157 | 0.693 | < .001 |
| Naming Type ✻ Stimulation Time ✻ Region ✻ Baseline Naming Speed | < .001 | 1 | < .001 | 0.003 | 0.953 | < .001 |
| Naming Type ✻ Stimulation Time ✻ Region ✻ Fluid Intelligence | < .001 | 1 | < .001 | 0.015 | 0.903 | < .001 |
| Residuals | 0.340 | 66 | 0.005 |  |  |  |
| Stimulation Type ✻ Naming Type ✻ Stimulation Time | 0.007 | 1 | 0.007 | 0.888 | 0.349 | 0.013 |
| Stimulation Type ✻ Naming Type ✻ Stimulation Time ✻ Region | < .001 | 1 | < .001 | 0.080 | 0.778 | < .001 |
| Stimulation Type ✻ Naming Type ✻ Stimulation Time ✻ Fluid Intelligence | 0.008 | 1 | 0.008 | 0.974 | 0.327 | 0.015 |
| Stimulation Type ✻ Naming Type ✻ Stimulation Time ✻ Baseline Naming Speed | 0.001 | 1 | 0.001 | 0.180 | 0.673 | 0.003 |
| Stimulation Type ✻ Naming Type ✻ Stimulation Time ✻ Region ✻ Baseline Naming Speed | < .001 | 1 | < .001 | 0.047 | 0.829 | < .001 |
| Stimulation Type ✻ Naming Type ✻ Stimulation Time ✻ Region ✻ Fluid Intelligence | 0.008 | 1 | 0.008 | 0.975 | 0.327 | 0.015 |
| Residuals | 0.542 | 66 | 0.008 |  |  |  |

*Note.*  Type III Sum of Square

**Between Subjects Effects**

| **Cases** | **Sum of Squares** | **df** | **Mean Square** | **F** | **p** | **η²p** |
| --- | --- | --- | --- | --- | --- | --- |
| Region | 0.001 | 1 | 0.001 | 0.008 | 0.930 | < .001 |
| Fluid Intelligence | 0.192 | 1 | 0.192 | 1.992 | 0.163 | 0.029 |
| Baseline Naming Speed | 7.253 | 1 | 7.253 | 75.401 | < .001 | 0.533 |
| Region ✻ Baseline Naming Speed | < .001 | 1 | < .001 | 0.004 | 0.948 | < .001 |
| Region ✻ Fluid Intelligence | < .001 | 1 | < .001 | < .001 | 0.987 | < .001 |
| Residuals | 6.349 | 66 | 0.096 |  |  |  |

*Note.*  Type III Sum of Square

Supplementary Results

Baseline naming speed predicted naming speed during the stimulation sessions, F(1,66)=75.40, *p* ¸.001, η²ₚ = 0.53, but this was not the case for fluid intelligence, F(1,66)=1.99, *p*=.16, η²ₚ = 0.03.

We identified a significant interaction between Naming type x Region x Baseline Naming Speed, F(1,66)= 4.67, *p*= .03, η²ₚ = 0.07. We calculated the interaction between Naming type x baseline naming at the left IFG and the left TPJ independently. At the left IFG the interaction was not significant, F(1,70)= 3.09, *p*= .08, η²ₚ = 0.002. At the left TPJ the interaction was significant, F(1,70)= 10.31, *p*= .002, η²ₚ = 0.008. We explored the correlation between baseline naming speed and object and action naming in the left TPJ group. The correlation between baseline naming speed and action naming speed, r(70)=0.73, p<.001 was stronger than for object naming, r(70)=0.62, p<.001.
