# Supplementary material for: Regionally specific picture naming benefits of focal tDCS are dependent on baseline performance in older adults
Source: GeroScience. 2025 May 10;47(6):6839–49. doi: 10.1007/s11357-025-01674-x (PMC12638570; doi:10.1007/s11357-025-01674-x)
Supplement: Supplementary file 4 — Supplementary file4 (DOCX 27 KB) [file 11357_2025_1674_MOESM4_ESM.docx]

# **Supplementary Section 4. Blinding, Mood, and Adverse Effects analyses**

# Blinding

We used a chi-squared test to investigate how participants' age group and stimulated region affected their ability to properly identify the active session, demonstrating the efficiency of blinding. The chi-squared test revealed a significant association between age group and blinding scores, χ²(1, N = 64) = 6.399, *p* = .011, suggesting that age group influenced participants' ability to maintain blinding integrity. Younger adults (24/32 correct at left IFG; 25/32 correct at left TPJ) were able to guess active sessions better than older adults (16/32 correct at left IFG; 18/32 correct at left TPJ). Blinding efficacy was comparable across the two stimulation sites, χ²(1, N = 64) = 0.256, *p* = .61.

# Positive Mood

We conducted a 2×2×2 repeated-measures ANOVA to determine whether Anodal and Sham sessions with older and younger adults at the left IFG and TPJ influenced VAMS positive mood scores. A significant stimulation effect was found, *F*(1, 140) = 4.508, *p* = .035, η²ₚ = 0.012. Participants reported a small but significant reduction in VAMS positive scores during the Anodal stimulation session (*M* = −9.996) compared to the Sham session (*M* = −2.461). No interactions were identified for Stimulation × Age Group, *F*(1, 140) = 0.014, *p* = .907, η²ₚ < 0.001; Stimulation × Region, *F*(1, 140) = 0.074, *p* = .785; or Stimulation × Age Group × Region, *F*(1, 140) = 3.511, *p* = .063, η²ₚ = 0.02. Change scores are presented in Table 2.

# Negative Mood

No effect of stimulation on VAMS negative scores was observed, *F*(1, 140) = 1.033, *p* =

.311, η²ₚ = 0.002. Stimulation also did not affect VAMS negative scores for the following interactions: Stimulation × Age Group, *F*(1, 140) = 0.031, *p* = .860, η²ₚ < 0.001; Stimulation × Region, *F*(1, 140) = 0.186, *p* = .667, η²ₚ = 0.001; or Stimulation × Age Group × Region, *F*(1, 140) = 0.063, *p* = .802, η²ₚ < 0.001. Change scores are presented in Table 2.

| **Table 3.** Change in mood scores for sham and anodal stimulation across regions and age groups . | | | | | | | | |
| --- | --- | --- | --- | --- | --- | --- | --- | --- |
|  |  | left IFG |  |  |  | left TPJ |  |  |
|  | Older Adults | | Younger Adults | | Older Adults | | Younger Adults | |
|  | Sham | Anodal | Sham | Anodal | Sham | Anodal | Sham | Anodal |
|  | Mean  (sd) | Mean  (sd) | Mean  (sd) | Mean  (sd) | Mean  (sd) | Mean  (sd) | Mean  (sd) | Mean  (sd) |
| Change in Positive Mood | -0.41  (41.22) | -0.75  (40.37) | 0.33  (32.81) | -12.75  (38.16) | -1.91  (39.57) | -17.82  (41.39) | -7.19  (15.98) | -8.66  (21.92) |
| Change in Negative Mood | -20.58  (41.29) | -12.65  (78.17) | -9.32  (50.36) | -16.35  (45.48) | -20.59  (42.05) | -25.27  (46.15) | -11  (30.47) | -11.72  (51.89) |
| left IFG = left inferior frontal gyrus; left TPJ = left temporoparietal junction; sd = standard deviation. | | | | | | | | |
|  | | | | | | |  |  |

# Adverse Effects

Total adverse effects were not significantly different between sham and active stimulation sessions, *F*(1, 140) = 3.283, *p* = .072, η²ₚ = 0.02. No interaction effects were identified for Stimulation × Age Group, *F*(1, 140) = 0.486, *p* = .487, η²ₚ = 0.003; Stimulation × Region, *F*(1, 140) = 0.002, *p* = .963, η²ₚ < 0.001; or Stimulation × Age Group × Region, *F*(1, 140) = 0.779, *p* = .379, η²ₚ = 0.006. All adverse effects are presented in Table 4.

| **Table 4.** Adverse effects across stimulation site, age group, and stimulation type | | | | | | | | |
| --- | --- | --- | --- | --- | --- | --- | --- | --- |
|  |  | Left IFG |  |  | Left TPJ | | | |
|  | Older Adults | | Younger Adults | | Older Adults | | Younger Adults | |
|  | Sham | Anodal | Sham | Anodal | Sham | Anodal | Sham | Anodal |
|  | Mean  (sd) | Mean  (sd) | Mean  (sd) | Mean  (sd) | Mean  (sd) | Mean  (sd) | Mean  (sd) | Mean  (sd) |
| Headache | 1  (0) | 1.06  (0.33) | 1.11  (0.32) | 1.17  (0.45) | 1.08  (0.28) | 1.06  (0.23) | 1.31  (0.52) | 1.20  (0.47) |
| Neck Pain | 1.05  (0.23) | 1.02  (0.17) | 1.06  (0.33) | 1.06  (0.23) | 1.06  (0.33) | 1.08  (0.37) | 1.03  (0.17) | 1.03  (0.17) |
| Scalp Pain | 1.03  (0.17) | 1.06  (0.23) | 1.14  (0.42) | 1.08  (0.29) | 1.06  (0.33) | 1.14  (0.42) | 1.03  (0.17) | 1.14  (0.42) |
| Tingling | 1.36  (0.49) | 1.08  (0.50) | 1.42  (0.65) | 1.61  (0.64) | 1.53  (0.70) | 1.55  (0.70) | 1.77  (0.77) | 1.97  (0.88) |
| Itching | 1.06  (0.23) | 1.03  (0.17) | 1.17  (0.51) | 1.19  (0.40) | 1.08  (0.37) | 1.14  (0.49) | 1.14  (0.35) | 1.25  (0.50) |
| Burning | 1  (0) | 1.06  (0.24) | 1.08  (0.37) | 1.25  (0.55) | 1.17  (0.61) | 1.11  (0.40) | 1.17  (0.38) | 1.28  (0.70) |
| Skin Redness | 1  (0) | 1  (0) | 1.05  (0.33) | 1  (0) | 1  (0) | 1  (0) | 1.03  (0.17) | 1.08  (0.28) |
| Sleepiness | 1.08  (0.28) | 1.06  (0.23) | 1.44  (0.61) | 1.50  (0.61) | 1.19  (0.40) | 1.14  (0.35) | 1.53  (0.74) | 1.47  (0.61) |
| Trouble Concentrating | 1.06  (0.23) | 1.14  (0.42) | 1.31  (0.52) | 1.19  (0.40) | 1.28  (0.51) | 1.25  (0.44) | 1.28  (0.45) | 1.33  (0.63) |
| Mood change | 1.03  (0.17) | 1.06  (0.23) | 1.06  (0.23) | 1.03  (0.17) | 1  (0) | 1  (0) | 1  (0) | 1.03  (0.17) |
| IFG = Inferior frontal gyrus; TPJ = Temporoparietal junction; sd = standard deviation | | | | | | | | |
